# Assessment of the Levels of Knowledge Regarding Cataract and Glaucoma in Saudi Arabia and Measurement of the Ability to Differentiate Between the Two

**DOI:** 10.7759/cureus.19849

**Published:** 2021-11-23

**Authors:** Abdulmajeed A Alammar, Abdulaziz M Alabdulkareem, Abdallah B Abu-amara, Hatem Kalantan

**Affiliations:** 1 Medicine, King Saud University, Riyadh, SAU; 2 Ophthalmology, King Saud University, Riyadh, SAU

**Keywords:** public, ophthalmology, knowledge, saudi arabia, glaucoma, cataract

## Abstract

Objectives

To assess the general public’s level of knowledge on glaucoma and cataract and measure their ability to differentiate between the two.

Materials and methods

This was an analytic, cross-sectional study. We used a self-explanatory questionnaire to obtain information regarding the level of knowledge of glaucoma and cataract and measured the ability of the public to differentiate between the two in Saudi Arabia. The obtained results were manually entered into an Excel sheet and analyzed using the Statistical Package for the Social Sciences (SPSS) software version 26.

Results

The levels of knowledge on glaucoma and cataract and those of education were significantly associated (chi-square: P < 0.001). There was a significant association between having an eye condition and the ability to correctly define glaucoma and cataract (chi-square: P = 0.002). Concerning the definition of glaucoma, 48.4% of the participants who had a previous eye disorder answered correctly, whereas 40.1% of the participants who had no previous eye disorder answered correctly. In addition, 20.9% of the participants with a previous eye disease and 17.6% of the participants without any previous eye disease defined glaucoma incorrectly as cataract. A total of 71.4% of the participants with a previous eye disease, compared with 49.6% of the participants without any previous eye disease, correctly defined cataract. In addition, only 7.3% of the participants with a history of eye disease answered the definition of cataract as that of glaucoma (glaucoma: chi-square, P = 0.002; cataract: chi-square, P < 0.001).

Conclusion

This study is in line with other studies measuring the knowledge of the two diseases, with glaucoma being less known than cataract. While many of the participants were able to define glaucoma and cataract, they had many difficulties identifying how they present and which symptom belonged to cataract and glaucoma. Glaucoma and cataract were confused by a number of participants especially in the case of glaucoma as more defined it as cataract rather than the opposite.

## Introduction

Cataract and glaucoma are common diseases with a steeply increasing incidence over the age of 60 years [1]; both conditions occur more frequently with advancing age. Cataract and glaucoma frequently coexist in our patient population [2]. They are serious conditions and can cause visual loss. As the average life expectancy increases globally, many diseases, such as glaucoma and cataract, are becoming more prevalent [3]. With glaucoma being a leading cause of blindness, increasing the population’s level of awareness regarding the disease is necessary. As the disease is treatable in the early stages, early diagnosis will be cost-effective and reduce overall blindness rates [4]. Cataract is also a common cause of blindness. Both diseases are caused by multifactorial risk factors [5]. We believe that the cause of confusion between the two diseases is their similar names in Arabic, which are white and blue water for cataract and glaucoma, respectively. In the clinic, we noticed that patients get confused between the two.

Genetic predisposition, smoking, diabetes mellitus, drug usage, and ultraviolet B exposure all are associated with cataract [5]. The most important risk factors for multiple types are age and heredity. While the inherited component is self-evident, advancing age acts as a proxy for a number of external risk factors, the cumulative effect of which is significant [6]. The treatment of cataract blindness remains a significant issue in developing countries where there is a lack of knowledge and harder access to surgical care [7].

Glaucoma affects tens of millions worldwide, and that is only expected to increase, making glaucoma an important public health issue [8]. Primary open-angle glaucoma is a type of glaucoma that causes progressive visual neuropathy. It is the most prevalent type. Early identification is critical as the disease is curable and the visual impairment it causes is irreversible [9]. Because it can go unnoticed until it is too late, diagnosis is commonly delayed [10]. The symptoms of the early-stage disease are probably minimal or nonexistent. It has several types, traditionally classified as primary or secondary open-angle glaucoma or angle-closure glaucoma [11].

Every treatment for progressive glaucomatous optic neuropathy has the potential for side effects and comes with a certain amount of risk and cost. The use of a topical selective or nonselective α-blocker or a topical prostaglandin analog is usually the first-line treatment for glaucoma; α-agonists and topical carbonic anhydrase inhibitors are the second line of treatment. Laser trabeculoplasty and incisional surgery are additional methods for lowering intraocular pressure in patients who do not respond to antiglaucoma medications [11].

According to the World Health Organization, the prevalence of visual impairment was 23.5%, whereas the prevalence of blindness was 1.7%; these are the highest estimated prevalence in Saudi Arabia. Cataract was the leading cause of impaired vision, followed closely by refractive error [12].

Evaluation of the difference between glaucoma and cataract in terms of knowledge has been done in Saudi Arabia, but not extensively. Measuring the confusion between the two on the other hand has not been researched previously. In a study in Riyadh in 2017, 14.8% of the participants were found to have acceptable levels of knowledge on glaucoma (14.8%) considering that they correctly answered at least 50% of questions asked [13]. A study in Tehran in 2014 compared the participants’ levels of knowledge on glaucoma and cataract and found that their levels of knowledge on glaucoma were significantly lower than that of cataract. Furthermore, 46% of the participants had heard about glaucoma, but only 19.2% could correctly define it, whereas 82.9% had heard about cataract, and 57% correctly defined it. In the same study, the level of knowledge of females on both conditions was better than that of males [14]. Furthermore, a study in the western region of Saudi Arabia showed that 75% of the participants did not know that cataract can lead to blindness [15], and a study in Hong Kong showed similar results as most participants did not know the symptoms of glaucoma [16].

Over the past three decades, the age-adjusted prevalence of blindness has reduced, yet progress is not keeping pace with needs due to population growth [17]. There are many causes of blindness, such as uncorrected refractive error [18], retinitis pigmentosa, optic atrophy [19], trachoma [20], and trauma [21]. However, glaucoma is the third most common cause of blindness globally, following cataract and trachoma [22].

Early diagnosis of cataract may reduce visual impairment and blindness [23]. Detection of glaucoma at earlier stages is vital in preventing its progression. The high prevalence and rate of blindness make glaucoma a public health concern [24]. Early detection and screening for cataract are important to prevent glaucoma, which can be mechanical when there is a pupillary blockage or phacolytic, which is characterized by signs and symptoms of acute glaucoma [25]. Therefore, in this study, we aimed to assess the levels of knowledge of the public on glaucoma and cataract and to measure the public’s ability to differentiate between the two. Confusion between the two might delay treatment or diagnosis for those with glaucoma as they may think what they have or are at risk of having is not an urgent and irreversible disease. To the best of our knowledge, this is the first study in the Kingdom of Saudi Arabia with this number of participants.

## Materials and methods

Study design and population

This is a cross-sectional, observational study with 953 individuals. The study population included people in Saudi Arabia recruited from August 1, 2021, to October 8, 2021. We included respondents older than 15 years of age. Individuals less than 15 years of age or patients with psychological problems, such as dementia and Alzheimer’s disease, were excluded.

Data collection

We used a self-explanatory, electronically developed questionnaire for eye disease in Arabic with four sections: personal information of the participants, medical information on eye disease, knowledge on differentiating between glaucoma and cataract (two questions), risk factors (two questions), systems of glaucoma and cataract (13 questions), and prognoses and treatment (six questions).

The online questionnaire that we developed was tested using a pilot study for validity and reliability on 138 subjects. Information on age, gender, educational level, city of residence, diabetes mellitus, and hypertension were also collected from each participant. The questionnaire was written in Arabic, and it was used to assess the level of knowledge of the participants (Tables 1, 2).

**Table 1:** Section I of the questionnaire (personal information of the participants).

S: No.:	Question	Response
1	Gender	a) Male
		b) Female
2	Age	________ years
3	Nationality	a) Saudi
		b) Non-Saudi
4	Educational level	a) Illiterate
		b) Elementary
		c) Intermediate
		d) Secondary
		e) Bachelors
		f) Graduate or postgraduate
5	In which city do you live?	a) Riyadh
		b) Jeddah
		c) Mecca
		d) Medina
		e) Al-Ahsa
		f) Dammam
		g) Taif
		h) Other: ________
6	Do you work in the health field?	a) Yes
		b) No
7	Have you visited an eye clinic before?	a) Yes
		b) No
8	Do you have any eye disease?	a) Yes
		b) No
		If the answer is yes, specify: ________
9	Do you have diabetes?	a) Yes
		b) No
10	Do you have hypertension?	a) Yes
		b) No
11	Is there any family history of glaucoma or cataract?	a) Yes
		b) No
12	Do you know someone with glaucoma or cataract?	a) Yes
		b) No

**Table 2:** Section II of the questionnaire (medical information on glaucoma and cataract of the participants).

S: No.:	Question	Response
1	Which of the following is true regarding glaucoma?	a) Opacification of the eye lens
		b) Optic nerve damage likely to be caused by increased pressure inside the eye
		c) Corneal inflammation
		d) Retinal detachment
		e) Excessive tearing
		f) I do not know
2	Which of the following is true regarding cataract?	a) Opacification of the eye lens
		b) Optic nerve damage likely to be caused by increased pressure inside the eye
		c) Corneal inflammation
		d) Retinal detachment
		e) Excessive tearing
		f) I do not know
3	Which of the following is a risk factor for glaucoma?	a) Family history
		b) Diabetes mellitus
		c) Smoking
		d) Cortisone use
		e) Excessive sun exposure
		f) Age above 60 years
		g) Use of contact lenses
		h) Severe myopia
		i) Dark skin
4	Which of the following is a risk factor for cataract?	a) Family history
		b) Diabetes mellitus
		c) Smoking
		d) Cortisone use
		e) Excessive sun exposure
		f) Age above 60 years
		g) Use of contact lenses
		h) Severe myopia
		i) Dark skin
5	Blind spots are a symptom of which of the following?	a) Glaucoma
		b) Cataract
		c) Glaucoma and cataract
		d) Neither
		e) I do not know
6	Impaired night vision is a symptom of which of the following?	a) Glaucoma
		b) Cataract
		c) Glaucoma and cataract
		d) Neither
		e) I do not know
7	Blurry vision is a symptom of which of the following?	a) Glaucoma
		b) Cataract
		c) Glaucoma and cataract
		d) Neither
		e) I do not know
8	Sensitivity to light and glare is a symptom of which of the following?	a) Glaucoma
		b) Cataract
		c) Glaucoma and cataract
		d) Neither
		e) I do not know
9	Tunnel vision is a symptom of which of the following?	a) Glaucoma
		b) Cataract
		c) Glaucoma and cataract
		d) Neither
		e) I do not know
10	A shadow covering the visual field is a symptom of which of the following?	a) Glaucoma
		b) Cataract
		c) Glaucoma and cataract
		d) Neither
		e) I do not know
11	Eye pain is a symptom of which of the following?	a) Glaucoma
		b) Cataract
		c) Glaucoma and cataract
		d) Neither
		e) I do not know
12	Seeing halos around lights is a symptom of which of the following?	a) Glaucoma
		b) Cataract
		c) Glaucoma and cataract
		d) Neither
		e) I do not know
13	Flashes of light are a symptom of which of the following?	a) Glaucoma
		b) Cataract
		c) Glaucoma and cataract
		d) Neither
		e) I do not know
14	Loss of vision for a few seconds is a symptom of which of the following?	a) Glaucoma
		b) Cataract
		c) Glaucoma and cataract
		d) Neither
		e) I do not know
15	Frequent change in the prescription of glasses is a symptom of which of the following?	a) Glaucoma
		b) Cataract
		c) Glaucoma and cataract
		d) Neither
		e) I do not know
16	Eye redness is a symptom of which of the following?	a) Glaucoma
		b) Cataract
		c) Glaucoma and cataract
		d) Neither
		e) I do not know
17	Excessive tearing is a symptom of which of the following?	a) Glaucoma
		b) Cataract
		c) Glaucoma and cataract
		d) Neither
		e) I do not know
18	Glaucoma may lead to blindness.	a) True
		b) False
		c) I do not know
19	Cataract may lead to blindness.	a) True
		b) False
		c) I do not know
20	Loss of vision due to glaucoma can be restored.	a) True
		b) False
		c) I do not know
21	Loss of vision due to cataract can be restored.	a) True
		b) False
		c) I do not know
22	Glaucoma can be treated with medication.	a) True
		b) False
		c) I do not know
23	Cataract can be treated with medication.	a) True
		b) False
		c) I do not know

Ethical considerations

Ethical approval for this study was granted by the Institutional Review Board (IRB), King Saud University (E-21-6090), and the College of Medicine Research Center (CMRC), Riyadh, Saudi Arabia. All the participants received a clear explanation about the study and signed informed consent. Individuals who refused to participate were excluded.

Statistical analysis

As the study used a descriptive approach, different statistical commands of medical statistics techniques were used. Analyses were performed using the Statistical Package for the Social Sciences (SPSS) software version 26 (IBM, SPSS Inc., Armonk, NY, USA). Appropriate statistical models and analyses were used according to the data type. Descriptive statistics such as frequency were used for tables and relevant variables, and percentages were used for variables on basic information such as age, gender, student's major, year of the study, and other related variables. Cross tabulation was used to create cross-tabulation tables (2 × 2) and multi-day tables (more than two rows and columns). The chi-square test was used to compare the results, and bar charts were used to clearly present some variable relationships. We verified the validity of the study tool through the internal consistency validity method as follows. For internal consistency validity, we calculated Pearson's correlation coefficient by presenting the score for each question that belongs to each axis and the total score for the axis to which it belongs.

## Results

Of the 953 respondents (41.4% male and 58.6% female) (Table 3), 783 individuals lived in Riyadh, Saudi Arabia. The average age of the participants was 32 years, 59% of the respondents had a bachelor's degree, and 27% of the respondents worked in the healthcare field. The presence of eye diseases was reported in 28.6% of the participants, 31% of participants had diabetes mellitus, and 22.8% of them had hypertension.

**Table 3:** Frequency distribution of personal information. Listing the type of eye disease was optional because only 103 of the 237 participants answered with the type of eye disease.

Variable	Category	n (%)
Gender	Male	395 (41.4)
	Female	558 (58.6)
Age	Average age	31.99
Level of education	Illiterate	20 (2.1)
	Elementary	7 (0.7)
	Intermediate	24 (2.5)
	Secondary	248 (26)
	Bachelors	560 (58.8)
	Graduate or postgraduate	94 (9.9)
City of residence	Riyadh	783 (82.2)
	Outside of Riyadh	170 (17.8)
Do you work in the healthcare field?	Yes	259 (27.2)
	No	694 (72.8)
Do you have diabetes?	Yes	299 (31.4)
	No	654 (68.8)
Do you have hypertension?	Yes	217 (22.8)
	No	736 (77.2)
Have you visited an eye clinic before?	Yes	573 (60.1)
	No	380 (39.9)
Do you have any eye diseases?	Yes	273 (28.6)
	No	680 (71.4)
Eye disease type	Refractive error	53 (5.5)
	Cataract	17 (1.7)
	Glaucoma	4 (0.4)
	Dry eye	6 (0.6)
	Other	23 (2.4)
Family history of glaucoma or cataract	Yes	444 (46.6)
	No	509 (53.4)
Knows someone with glaucoma or cataract	Yes	550 (57.7)
	No	403 (42.3)
Total		953 (100)

Glaucoma was correctly defined by 42.5% of the participants, and cataract was correctly defined by 55.8% of the participants (Table 4). A total of 18.6% of the participants defined glaucoma incorrectly as cataract, and 8.8% answered the definition of cataract as that of glaucoma. Moreover, 26% and 23% answered “I do not know” for glaucoma and cataract, respectively.

**Table 4:** Frequency distribution of the participants' ability to define glaucoma and cataract. Each column is a separate question followed by the choices as rows. *IOP: intraocular pressure

	Glaucoma	Cataract
Definition	n (%)	n (%)
Opacification of the eye lens	177 (18.6)	532 (55.8)
Optic nerve damage likely to be caused by increased IOP*	405 (42.5)	84 (8.8)
Corneal inflammation	63 (6.6)	45 (4.7)
Retinal detachment	37 (3.9)	46 (4.8)
Excessive tearing	23 (2.4)	27 (2.8)
I do not know	248 (26)	219 (23)
Total	953 (100)	953 (100)

**Figure 1:**
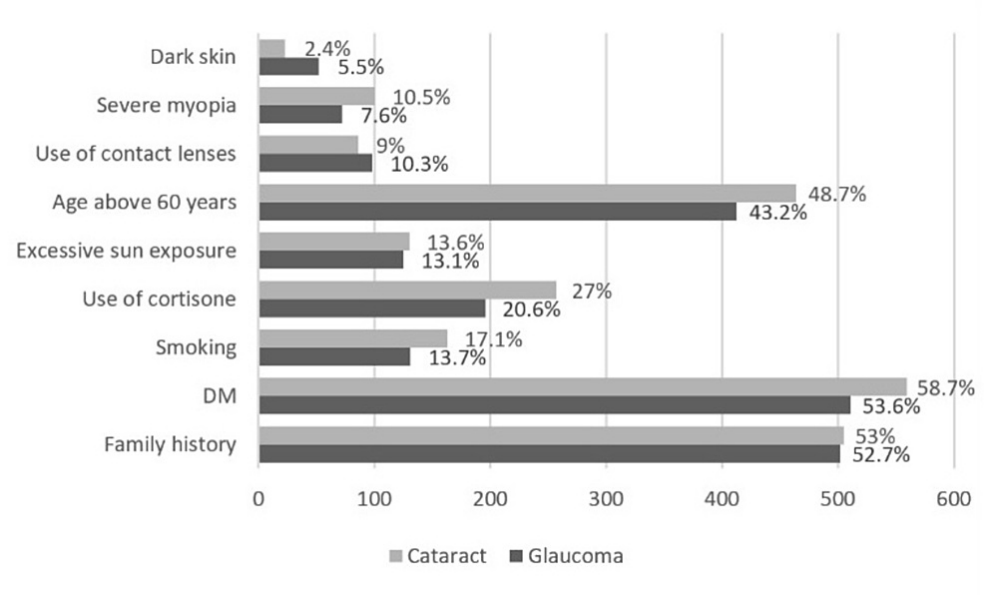
Frequency distribution of the risk factors for glaucoma and cataract. The participants were able to choose more than one risk factor.

Furthermore, 502 (52.7%), 511 (53.6%), and 412 (43.2%) of the respondents indicated that family history, diabetes mellitus, and age above 60 years, respectively, were risk factors for glaucoma, and 53%, 58.7%, and 48.7%, respectively, indicated that they were risk factors for cataract (Figure 1). Moreover, severe myopia was chosen as a risk factor for glaucoma and cataract by 7.6% and 10.5% of the respondents, respectively, while smoking was chosen as a risk factor for glaucoma and cataract by 13.7% and 17.1% of the respondents, respectively.

Concerning the knowledge on the symptoms, eye pain, redness, and seeing halos around lights were chosen by 26.8%, 20%, and 19.5% of the respondents as symptoms of glaucoma, respectively, and 43.7% of the participants answered that cataract can cause blurry vision. Furthermore, 21.7% answered that cataract can cause frequent changes in the prescription of glasses (Table 5).

**Table 5:** Frequency distribution of the symptoms of glaucoma and cataract. Columns are what the participants answered for each symptom. Each symptom was a separate question.

	Glaucoma	Cataract	Both	Neither	I do not know
Symptom	%	%	%	%	%
Blind spots	21.5	22.6	19.0	3.9	33.0
Impaired night vision	13.4	35.9	16.9	4.2	29.6
Blurry vision	14.1	43.7	17.4	1.5	23.4
Sensitivity to light and glare	20.7	30.0	14.8	2.2	32.3
Tunnel vision	23.3	18.8	15.5	3.0	39.3
A shadow covering the visual field	21.8	21.3	21.3	3.0	32.5
Eye pain	26.8	13.4	22.6	6.9	30.3
Seeing halos around lights	19.5	22.5	19.0	4.0	35.0
Flashes of light	17.2	21.3	19.2	4.5	37.8
Loss of vision for a few seconds	19.7	19.2	21.4	4.0	35.7
Frequent changes in the prescription of glasses	15.8	21.7	19.3	6.2	36.9
Eye redness	20.0	16.3	24.6	8.9	30.2
Excessive tearing	21.2	18.9	20.8	6.6	32.5

Over half (56.9%) of the participants responded that glaucoma could lead to blindness, whereas 40% said that cataract can lead to blindness. Additionally, 37% and 48.2% of the participants answered that loss of vision due to glaucoma and cataract can be restored, respectively, and 38.4% and 36.7% of the respondents stated that glaucoma and cataract, respectively, could be treated with medications (Table 6).

**Table 6:** Frequency distribution of the prognosis and treatment questions. The first two variables are concerned with the prognosis, whereas the last four variables are concerned with the treatment.

	True	False	I do not know
Variable	n (%)	n (%)	n (%)
Glaucoma may lead to blindness.	542 (56.9)	181 (19.0)	230 (24.1)
Cataract may lead to blindness.	385 (40)	342 (35.9)	226 (23.7)
Loss of vision due to glaucoma can be restored.	355 (37.3)	286 (30.0)	312 (32.7)
Loss of vision due to cataract can be restored.	459 (48.2)	192 (20.0)	302 (31.7)
Glaucoma can be treated with medication.	366 (38.4)	291 (30.5)	296 (31.1)
Cataract can be treated with medication.	350 (36.7)	323 (33.9)	280 (29.4)

There was a significant association between overall knowledge on glaucoma and cataract, and educational level (chi-square: P < 0.001). Intermediate school graduates (41.7%), undergraduates (46.4%), and graduates (55.3%) correctly defined glaucoma, which is optic nerve damage due to high intraocular pressure. In addition, secondary school graduates (26.2%), undergraduates (25.9%), and graduates (17%) answered “I do not know.” Moreover, 42.7%, 60.4%, and 75.5% of the secondary school graduates, undergraduates, and graduates, respectively, correctly defined cataract. There did not seem to be a significant relationship between education and knowledge of risk factors, except in a few cases, such as diabetes; 74.5% of postgraduates knew that it is a risk factor, whereas only 61.8% of undergraduates did.

A total of 75% of healthcare workers correctly defined glaucoma compared with 30.4% of those who were not from the healthcare field. Moreover, 83% of the healthcare workers correctly defined cataract compared with 45.7% of the participants from other fields. Only 5.8% and 3.5% of the healthcare workers answered “I do not know” for the definition of glaucoma and cataract, respectively, compared with 33.6% and 30.3% in those from other fields, respectively (P < 0.001).

Table 7 shows the level of knowledge on glaucoma and cataract in participants with previous eye disease. Regarding glaucoma definition, 48.4% of the participants with previous eye disease answered correctly compared with 40.1% without previous eye disease who answered correctly. Furthermore, 20.9% individuals with previous eye disease answered the definition of cataract as the definition of glaucoma compared with 17.6% without eye disease who did the same. As for the definition of cataract, 71.4% of those with eye disease answered correctly compared with 49.6% of those without eye disease (chi-square: P = 0.002 and P < 0.001, respectively).

**Table 7:** Cross tabulation of having eye disease across glaucoma and cataract definition. *P-value has been calculated using chi-square test, P = 0.002 and P < 0.001 for glaucoma and cataract, respectively. § Significant at P < 0.05 **IOP: intraocular pressure

			Eye disease/disorder		Eye disease/disorder				
	P -value*	Definition	Yes	No	No	Yes	Definition	P-value*	
			n (%)	n (%)	n (%)	n (%)			
Glaucoma	0.002§	Opacification of the eye lens	57 (20.9)	120 (17.6)	337 (49.6)	195 (71.4)	Opacification of the eye lens	0.001§	Cataract
		Optic nerve damage likely to be caused by Increased IOP	132 (48.4)	273 (40.1)	64 (9.4)	20 (7.3)	Optic nerve damage likely to be caused by Increased IOP**		
		Corneal inflammation	18 (6.6)	45 (6.6)	30 (4.4)	15 (5.5)	Corneal inflammation		
		Retinal detachment	15 (5.5)	22 (3.2)	35 (5.1)	11 (4.0)	Retinal detachment		
		Excessive tearing	4 (1.5)	19 (2.8)	25 (3.7)	2 (0.7)	Excessive tearing		
		I do not know	47 (17.2)	201 (29.6)	189 (27.8)	30 (11.0)	I do not know		
		Total	273(28.6)	680 (71.4)	680 (71.4)	273 (28.6)	Total		

## Discussion

The levels of knowledge on the two diseases were medium, with cataract being expectedly more known than glaucoma. While many could define the two diseases, few knew how they present as the participants faced many difficulties identifying which symptom belonged to cataract and glaucoma. Disease presentation is an essential part of preventing further progression, especially in the case of glaucoma, regarding which the respondents were less informed. Well-known risk factors such as diabetes, age above 60 years, and family history were correctly answered by over half of the participants, whereas in comparison, lesser-known risk factors such as dark skin, severe myopia, and sun exposure had significantly less correct answers. Both glaucoma and cataract can lead to blindness, but while 56.9% of the participants agreed that glaucoma can cause it, only 40% knew that cataract can lead to blindness. Healthcare workers expectedly did much better than others, and there also did not seem to be much confusion among them as very few answered that they did not know the definition of the two diseases.

Another part of the study was trying to assess whether there was confusion between the two diseases as they have similar names, i.e., white water and blue water for cataract and glaucoma, respectively, and we wanted to know whether people truly get confused between the two as observed in our daily practice. Therefore, we tested this by comparing the number of those that answered the definition of glaucoma as that of cataract and the other way around. A considerable number of participants got confused between the two, especially glaucoma, and 18.6% of the participants answered that glaucoma was opacification of the eye lens, and while this is significant, we expected more confusion as both have similar names.

Our results are consistent with the results of several studies showing that knowledge about cataract is more than that of glaucoma while also highlighting that knowing the disease definition does not translate to knowing the symptoms [14,16]. In the present study, 42.5% of the participants correctly defined glaucoma compared to 19.2% in a study in Iran, albeit knowing the definition of cataract was consistent with the same study as 55.8% of our participants answered it correctly in comparison to 57% that knew the definition in the Iranian study [14]. The present study had limitations, one of which was that as the questionnaire was an online questionnaire, the participants were limited to those who could be connected to the Internet and relatively younger population that may be more educated. Another limitation is the use of the same choices on being asked about the definitions for glaucoma and cataract as the participants were presented with the same choices that might have made it easier to answer. Although it is a limitation, it was important to have it so that we can determine whether there was any confusion between the two diseases. Despite the limitations, to the best of our knowledge, there are no previous studies with such a sample size in Saudi Arabia that investigate the knowledge on the two diseases and the confusion between them among the public. A notable finding of our study is that although other similar studies have shown that females had higher levels of knowledge, in our study, the differences were negligible and not significant [13,14].

## Conclusions

In conclusion, although many could define glaucoma and cataract, few could differentiate between their symptoms. This study also highlighted that although there are higher levels of knowledge regarding cataract than glaucoma in terms of definition, the higher levels of knowledge regarding the latter are necessary because of its irreversible effects. Our findings show that symptoms are less known and need to be focused on when educating the masses about eye diseases as the definition alone is insufficient. Some of the respondents were confused between glaucoma and cataract, as demonstrated by their answers. As glaucoma and cataract have similar names in Arabic, using other names that are also known might be a better alternative for physicians and health educators to lessen the confusion. Health educators and awareness campaigns need to focus more on the symptoms of both diseases, educate the masses on how they present, and target higher-risk populations such as people with diabetes to advise them to have annual eye examinations. Focused research on the confusion between the two diseases and the demographics will be beneficial for health educators and is a point for future research. Further information needs to be collected regarding the sources of information for eye diseases and assessment of people's levels of knowledge in terms of treatment. 
